# The role of CTLA-4 in immune regulation

**DOI:** 10.1016/j.imlet.2007.10.010

**Published:** 2008-01-15

**Authors:** Johan Verhagen, Catherine A. Sabatos, David C. Wraith

**Affiliations:** Department of Cellular and Molecular Medicine, School of Medical Sciences, University of Bristol, University Walk, Bristol BS8 1TD, UK

**Keywords:** CTLA-4, Immune regulation

The role of cytotoxic T-lymphocyte-associated antigen-4 (CTLA-4; CD152) in the regulation of T cell activation remains a much debated topic. A type I transmembrane protein of the immunoglobulin superfamily and homologue of the co-stimulatory molecule CD28, CTLA-4 serves a crucial role in negative regulation of the T cell immune response. Mice deficient in CTLA-4 develop spontaneous lymphoproliferative disease with multiorgan infiltration within 3–4 weeks of birth, suggesting aberrant regulation of T cell activation or proliferation. Several theories have been postulated to explain the observed manifestation of spontaneous disease. First, the observed dysregulation could result from an impaired function of T regulatory (Treg) cells, since CTLA-4 has been shown to be constitutively expressed on the surface and in the cytoplasm of peripheral CD4^+^CD25^+^ Treg cells [1]. Several studies using neutralizing monoclonal antibody to block CTLA-4 on Treg cells *in vivo* have reported an exacerbation of autoimmune disease [1,2]. In contrast to these findings, B7-1/B7-2/CTLA-4-deficient mice showed FoxP3 expression on 2.5–3% of CD4^+^ cells, representing Treg cells that are functional *in vitro* [3]. The importance of CTLA-4 for Treg cell function therefore remains to be elucidated.

A second explanation for the augmented lymphoproliferation observed in CTLA-4^−/−^ mice could be a lower activation threshold in CTLA-4 deficient effector T cells. CTLA-4 has been suggested to regulate the threshold for activation in response to endogenous rather than exogenous antigens [4,5]. CTLA-4 has further been suggested to play a role in the negative selection of T cells in the thymus [6,7]. A deficiency in CTLA-4 might therefore give rise to a hyperresponsive T cell population, leading to disproportionate T cell proliferation and organ infiltration.

The current study elaborates on an altogether new explanation for the observed lymphoproliferation. In recent studies, the Rudd group have made several interesting observations regarding the effect of CTLA-4 on T cell motility. First, CTLA-4 ligation was shown to upregulate the expression of lymphocyte function-associated antigen-1 (LFA-1) [8]. LFA-1 plays a crucial role in promoting clustering of T cells with antigen presenting cells (APCs), therefore facilitating antigen presentation and activation. Interestingly, anti-CD3/CTLA-4 co-ligation simultaneously led to the inhibition of IL-2 production. Moreover, hyperactivation of adhesion is often accompanied by increased motility and migration of T cells, which could limit the number of engaged TCRs and therefore limit the antigen-specific response. The same stimulation could therefore lead to either a positive or negative response, depending on factors such as duration and combined strength of signalling.

The importance of signal duration in addition to quality and quantity was further supported by findings on the effect of CTLA-4 on the TCR stop signal [9]. Comparison of the mobility of CTLA-4^+/+^ and CTLA-4^−/−^ CD4^+^ cells on plates coated with the LFA-1 ligand, intercellular adhesion molecule-1 (ICAM-1), showed that whereas pre-activated T cells from either group migrated at similar speeds, treatment with anti-CTLA-4 led to the increased motility of CTLA-4^+/+^ but not CTLA-4^−/−^ cells. Further experiments demonstrated that while anti-CD3 ligation resulted in reduced movement of primary human and mouse T cells, co-ligation of CTLA-4 reversed the arrest. The authors argued that this event could play a role in secondary immune responses where shorter dwell times are sufficient for activation. CTLA-4^+^ cells, with shorter dwell times due to the reversal of the TCR stop signal, could therefore compete with CTLA-4^−^ cells which engage in longer conjugation with APCs. In addition, the shorter dwell time of CTLA-4^+^ may stop these cells from being activated by autoantigens, which would lead to the autoimmune disease commonly observed in CTLA-4 deficient mice.

In the current paper by Downey et al., the investigation into the effect on T cell migration is taken one step further by looking at CTLA-4 deficient mice with established lymphoproliferative disease. Whereas CTLA-4 ligation on pre-activated T cells from healthy mice and humans in the previous study led to reversal of the anti-CD3 induced TCR stop signal, CTLA-4^−/−^ T cells from diseased mice are now shown to have resistance to the induction of a TCR stop signal by anti-CD3 ligation. It is therefore argued that in diseased animals, the TCR is decoupled from its normal ability to induce a stop signal, resulting in the tissue infiltration observed in CTLA-4 deficient mice. Whether the decoupling of the TCR stop signal is the result of the chronic illness in diseased CTLA-4^−/−^ mice or arises from a difference in the thymic selection of T cells in the absence of CTLA-4 remains unclear. A comparison between the motility of T cells from CTLA-4^−/−^ animals before and after the manifestation of disease may shed some light on this matter.

The findings of the current study and the two previous studies by the same group [8,9] may at first seem at odds with each other. It is clear, however, that these studies used different conditions and sources of cells, which could explain the disparity of results. Nevertheless, taken together they demonstrate the delicate balance required for T cell activation and regulation in order to achieve a healthy immune response. CTLA-4 seems to play a pivotal role in controlling each part of the three-way balance between the strength, quality and duration of signal which determines the outcome of an interaction between a T cell and an APC. Exactly how CTLA-4 exerts its multidisciplinary effect remains unclear, but may prove crucial in the understanding of immune regulation.
